# Case Report: Spinal epidural lipomatosis with incomplete cauda equina syndrome treated with unilateral biportal endoscopic technique

**DOI:** 10.3389/fsurg.2026.1762865

**Published:** 2026-03-10

**Authors:** Zaiyin Deng, Yujin Wang, Mohammed Saud Shaik, Duanyang Li, Rongjing Di, Zhourui Wu, Bin Ma

**Affiliations:** 1 Division of Spine, Department of Orthopedics, Tongji Hospital affiliated to Tongji University, School of Medicine, Tongji University, Shanghai, China; 2 Key Laboratory of Spine and Spinal Cord Injury Repair and Regeneration, Ministry of Education of the People's Republic of China, Tongji University, Shanghai, China

**Keywords:** spinal epidural lipomatosis, cauda equina syndrome, unilateral biportal endoscopy, minimally invasive spine surgery, epidural fat, spinal stenosis

## Abstract

Spinal epidural lipomatosis (SEL) involves the aberrant growth of normal adipose tissue in the spinal canal. It lacks of distinctive clinical manifestations. However, it can infrequently present as incomplete cauda equina syndrome (CES), a condition necessitating urgent surgical intervention. We present a case of SEL primarily manifesting as acute incomplete CES. The patient underwent unilateral biportal endoscopic (UBE) spinal canal decompression and adipose tissue excision. This procedure led to satisfactory postoperative neurological improvement. This case underscores the efficacy of unilateral biportal endoscopy as a viable minimally invasive surgical approach for SEL when accompanied by incomplete CES.

## 1 Introduction

Spinal epidural lipomatosis, or SEL features excessive accumulation and aberrant proliferation of adipose tissue within the epidural space. This tissue situate around the spinal cord within the spinal canal and can compress the spinal cord or nerve roots (1). Although its overall prevalence is approximately 2.5%, only about 0.1% of cases present with specific symptoms (2).

Patients show various symptoms, including back pain, radicular pain, and motor weakness. Severe cases may involve lower urinary tract or anorectal dysfunction, known as cauda equina syndrome (CES) (3). Clinically, CES is classified into incomplete CES and complete CES. Incomplete CES presents with sensory or motor deficits but preserved voluntary bladder control. In contrast complete CES involves painless urinary retention and overflow incontinence (4). Incomplete CES is an infrequent but critical sequela of SEL. This condition has recently garnered escalating clinical attention. Current evidence suggests a robust correlation between SEL and idiopathic incomplete CES. Comorbid factors like adiposity potentially exacerbate their concurrence (5, 6). However, SEL remains rare and its symptoms are nonspecific. Clinicians often prioritize common causes like disc herniation or stenosis during incomplete CES emergencies. This clinical prioritization pattern may culminate in diagnostic dilemmas and delayed identification of SEL-induced incomplete CES.

Conservative management is the first-line intervention for mild to moderate SEL (3). Surgical intervention is imperative when SEL causes incomplete CES. Surgery alleviates neural compression and preserves neurological function (1, 7). This article presents a detailed case report of SEL manifesting primarily as acute incomplete CES. In the present case, we successfully excised the proliferated adipose tissue and accomplished spinal canal decompression using the unilateral biportal endoscopic (UBE) technique. Through the analysis of this case, this paper endeavors to comprehensively explore the key diagnostic criteria and surgical management strategies for SEL-related incomplete CES. Our aim is to enhance clinicians' diagnostic acumen and therapeutic efficacy in addressing this pathological condition.

## 2 Case presentation

### 2.1 History and physical examination

A 70-year-old male presented to our department with a one-month history of progressive perineal pain. This was accompanied by impaired light touch sensation in the same region. He denied urinary or bowel dysfunction, consistent with a diagnosis of incomplete CES. Three months ago, the patient had a prostatectomy for adenocarcinoma. He recovered well. He reported chronic steroid use but denied endocrine disorders.

**Figure 1:**
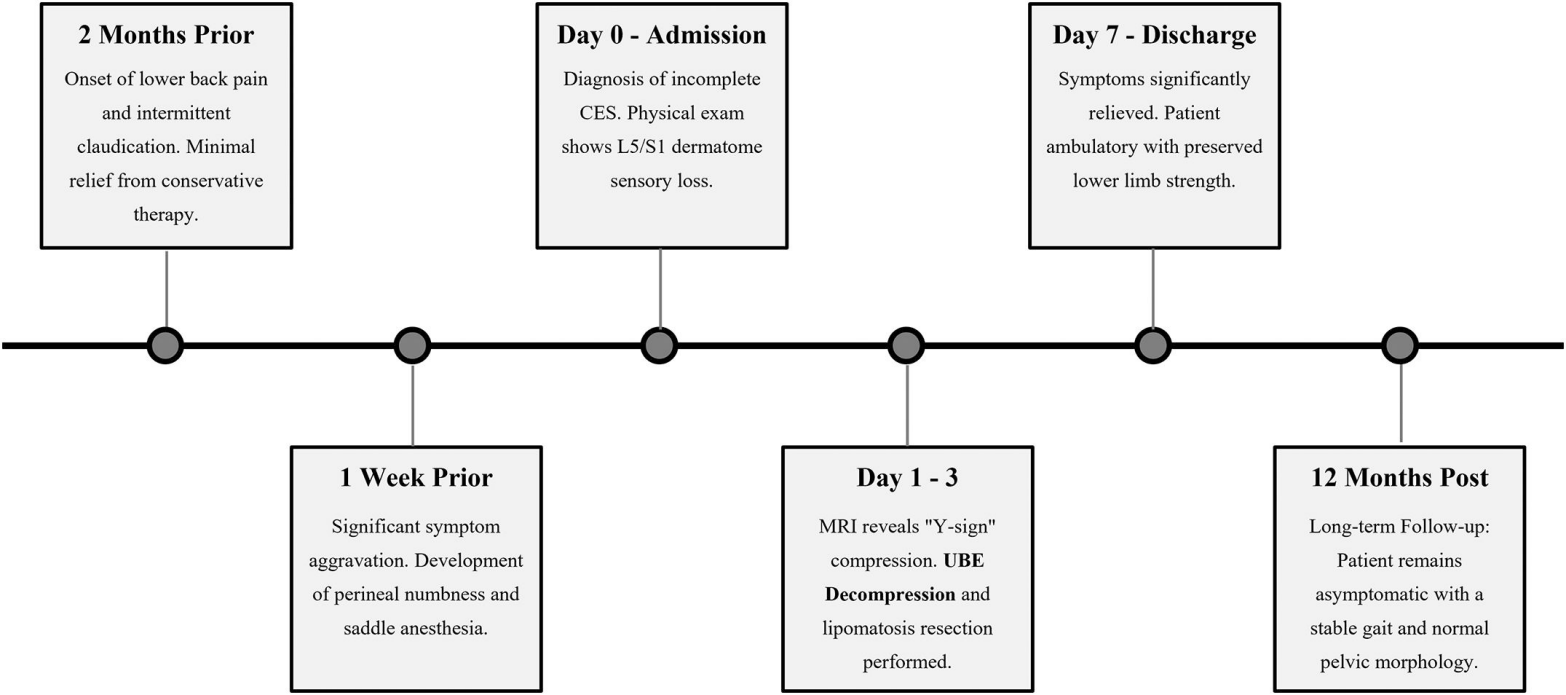
Timeline of clinical events and interventions. The timeline illustrates the patient's clinical course from the onset of symptoms (2 months prior) to the long-term follow-up (12 months post-surgery). Key events include the acute deterioration resulting in incomplete cauda equina syndrome (CES-I), the pathognomonic radiological diagnosis, the successful surgical intervention using the unilateral biportal endoscopic (UBE) technique, and the sustained neurological recovery.

Upon admission, his physical examination revealed a body mass index (BMI) of 29.4 kg/m^2^. Neurological exam showed tenderness and decreased pinprick sensation in the perineum. We found grade 5 muscle strength in all lower extremity groups. Bilateral pathological reflexes, including Lasegue and Bragard signs, were negative. We assessed the patient using the visual analog scale (VAS) and oswestry disability index (ODI). His scores were 7% and 74%, respectively. The timeline of the episode of care is summarized in (Figure 1).

### 2.2 Imaging findings

**Figure 2:**
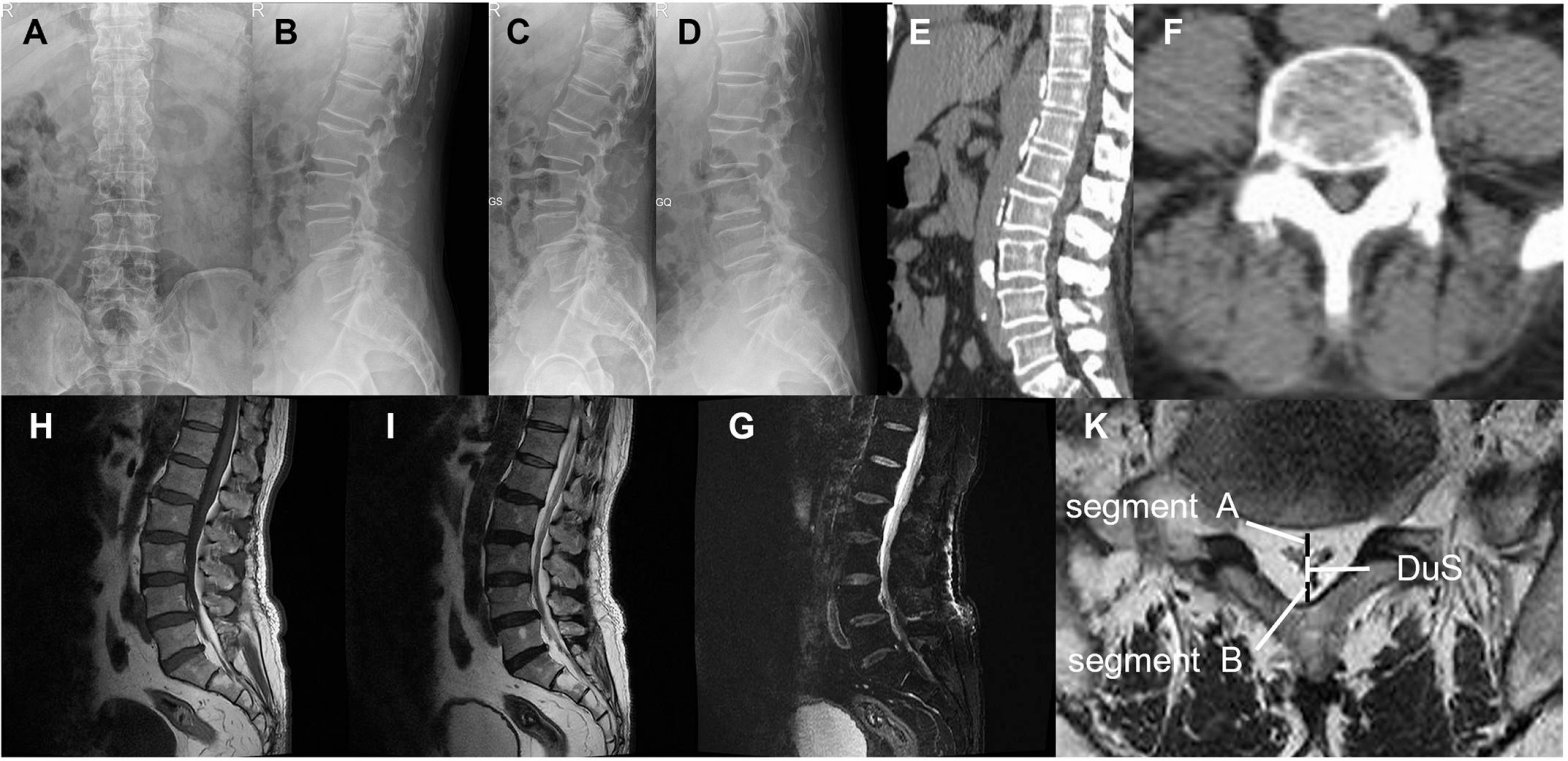
Preoperative radiological assessment. **(A,B)** Anteroposterior and lateral views reveal spondylolisthesis (slight retrolisthesis of L1 and anterolisthesis of L4) and osteophytosis from L1 to L5, with preserved intervertebral disc height and physiological curvature. **(C,D)** Flexion-extension radiographs demonstrate limited range of motion, indicating dynamic instability. **(E,F)** Preoperative sagittal **(E)** and axial **(F)** CT images show spinal canal stenosis and thecal sac compression at L5–S1. **(G–I)** Preoperative MRI (1.5 T, slice thickness 3 mm). Sagittal **(G)** and axial **(I)** views reveal significant accumulation of epidural adipose tissue (the “Y-sign”) extending from L3 to S1, causing marked circumferential compression of the thecal sac. Schematic of quantitative assessment: DuS (dural sac diameter), Segment A (ventral epidural fat), Segment B (dorsal epidural fat), Spi C (spinal canal diameter = DuS + A + B).

Laboratory analysis showed a white blood cell count of 10.07 × 10⁹/L and a neutrophil count of 8.82 × 10⁹/L (87.6%). The C-reactive protein level was elevated at 14.5 mg/L. Prostate-specific antigen levels were within the reference range. Lumbar flexion-extension x-rays revealed limited mobility (Figures 2A–D). Preoperative computed tomography (CT) showed thecal sac compression at L5-S1. Hypertrophic adipose tissue appeared as hypodense areas. These areas narrowed the spinal canal (Figures 2E,F). Lumbar magnetic resonance imaging (MRI) revealed a significant accumulation of epidural adipose tissue extending from L3 to S1. The tissue showed high signal intensity on T1 and T2 sequences. It caused marked compression of the thecal sac (Figures 2H,I). Axial views displayed the characteristic “Y sign” of thecal sac compression (8). The dural sac (DuS) to epidural fat (EF) ratio was 0.57, and the EF to spinal canal ratio was 64%. These findings were consistent with Borré grade II spinal epidural lipomatosis (9). We also identified concurrent multilevel intervertebral disc herniation and lumbar spondylolisthesis. Whole-body bone scintigraphy showed no evidence of osseous metastases.

### 2.3 Diagnostic assessment diagnostic methods

We established the diagnosis based on concordant clinical and radiological findings. The primary challenge was differentiating symptoms between L4 spondylolisthesis and SEL. Both conditions can cause mechanical compression. Given that the patient's saddle anesthesia and radicular pain strictly corresponded to the sacral nerve roots compressed by the adipose tissue at L5–S1, rather than the L4 level, SEL was identified as the primary culprit. Key differential diagnoses included lumbar disc herniation (LDH), spinal stenosis (LSS), and epidural neoplasms,such as angiolipoma and liposarcoma. LDH was excluded due to the absence of ventral disc material on MRI. Epidural neoplasms were ruled out based on the characteristic “Y-sign” and circumferential distribution of fat on MRI, distinct from the focal masses seen in tumors. This was later confirmed by histopathological analysis revealing benign mature adipocytes without vascular proliferation or atypia. The patient was diagnosed with incomplete CES as he retained voluntary bladder control. The prognosis for incomplete CES is generally favorable provided that surgical decompression is performed before the onset of urinary retention. Therefore, the prognostic characteristic for this patient was considered positive, contingent upon urgent surgical intervention.

### 2.4 Surgical intervention

**Figure 3:**
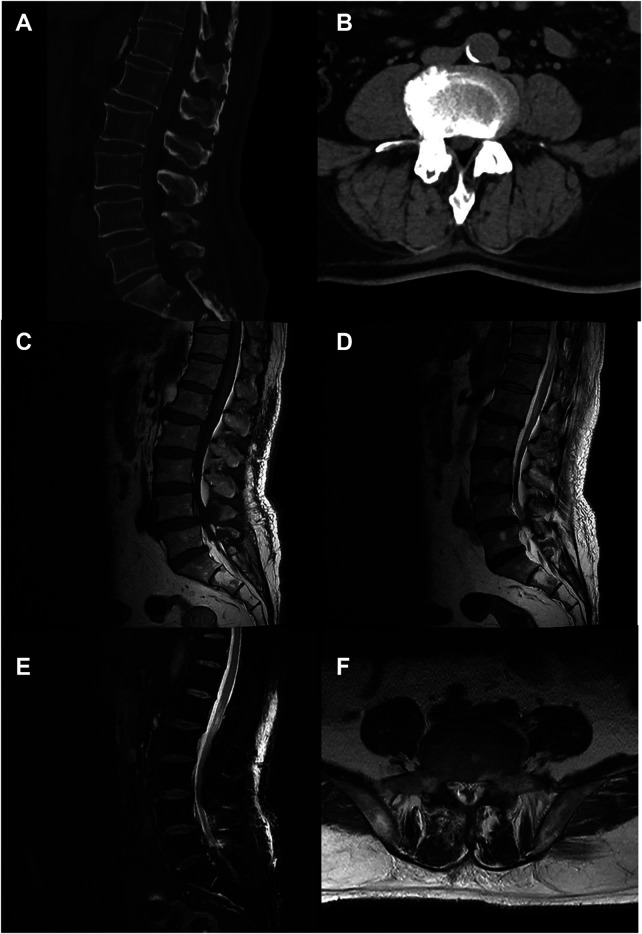
Intraoperative findings and histopathological examination. **(A,B)** Intraoperative fluoroscopy confirming the establishment of working portals at the L5–S1 level. **(C)** Endoscopic view showing the decompressed dura mater after complete resection of the hypertrophic epidural fat. **(D)** Macroscopic specimen of the excised tissue, showing lobulated yellow adipose tissue. **(E)** Low-power panoramic histopathological view (H&E stain, original magnification ×0.8) showing the overall architecture of the excised adipose tissue. **(F)** Higher magnification view (H&E stain, original magnification ×3.5) revealing mature adipocytes within a fibrovascular stroma, confirming the diagnosis of benign epidural lipomatosis.

Considering the severe neurological symptoms and imaging findings, surgical intervention was scheduled. Under general anesthesia, the L5/S1 levels were localized using C-arm fluoroscopy. Two working portals were established on the right side of the L5/S1 segments (Figures 3A,B). We performed precise laminectomy and decompression. Then, we resected the ligamentum flavum to expose the dural sac and nerve roots. Intraoperative observations confirmed hyperplasia of the epidural adipose tissue with rich capillary vasculature, resulting in circumferential compression. The hyperplastic fat tissue was thoroughly excised (Figures 3C,D). Following decompression, the restoration of dural sac pulsations was observed. After verifying hemostasis, 500 µg of mecobalamin and 40 mg of triamcinolone acetonide were administered locally to facilitate nerve function recovery. The incision was closed in layers over a gelatin sponge. The estimated blood loss was approximately 20 mL. Histopathological analysis revealed mature adipocytes with fibrovascular stroma, confirming benign epidural lipomatosis (Figures 3E,F). We found no diagnostic features of angiolipoma such as proliferating capillary vessels. Similarly, the specimen lacked lipoblasts or nuclear atypia characteristic of liposarcoma (10). We confirmed the diagnosis of benign Spinal Epidural Lipomatosis (SEL) based on the strict correlation between the diffuse radiological “Y-sign” and the classic histomorphology (11).

### 2.5 Follow-up and outcomes

**Figure 4:**
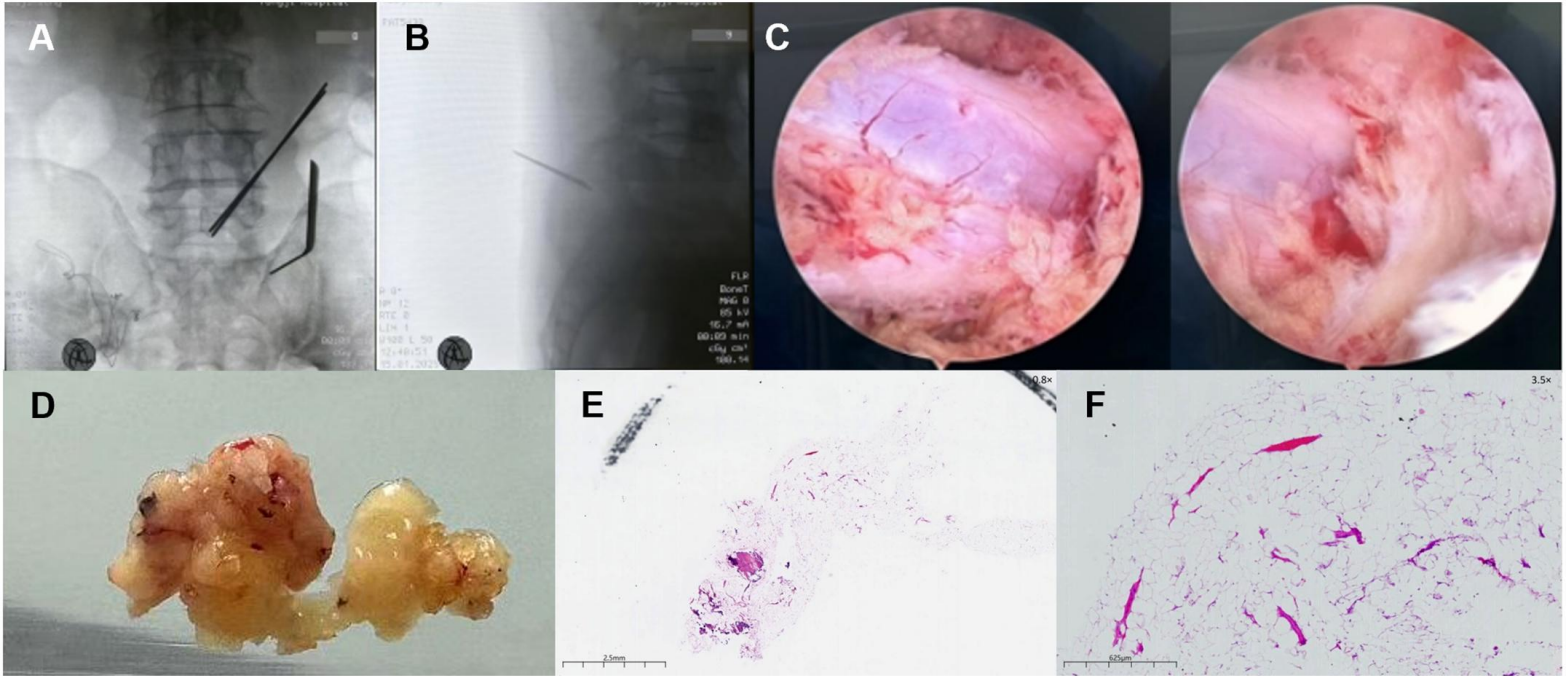
Postoperative imaging confirming effective decompression. **(A,B)** Postoperative CT images (sagittal and axial views) demonstrate adequate bony decompression at L5–S1. **(C–E)** Postoperative sagittal MRI (T1, T2, and fat-suppressed sequences) showing resolution of the epidural fat compression. Note the signal changes in the posterior soft tissue corresponding to the surgical tract. **(F)** Postoperative axial T2-weighted image confirms the restored patency of the spinal canal.

Postoperatively, the patient's recovery was uneventful. His vital signs remained stable, and he had no complications such as infection. He received routine postoperative care. CT and MRI performed on the third postoperative day confirmed satisfactory decompression of the thecal sac and resolution of epidural fat compression (Figure 4). Neurological examination at discharge revealed preserved muscle strength in both lower limbs. Perineal pain and paresthesia were notably diminished compared to admission. The patient was discharged on the fifth postoperative day.

At the 6-month follow-up, the patient exhibited significant neurological recovery. Perineal sensory abnormalities had essentially resolved, and he demonstrated unrestricted ambulation. The VAS score improved to 1, and the ODI score decreased to 18%. The patient reported satisfaction with the surgical outcome and significant improvement in quality of life.

At the 12-month follow-up, the patient remained entirely asymptomatic. Clinical evaluation confirmed the sustained resolution of perineal numbness and a stable, pain-free gait. This provides evidence of sustained cauda equina function recovery.

## 3 Discussion

In this case, we managed a rare presentation of acute incomplete CES caused by SEL using the UBE technique. The patient achieved rapid neurological recovery. His ODI improved from 74% to 18% at 6 months. This case demonstrates that UBE is a reliable salvage strategy for acute surgical emergencies. It provides adequate decompression while minimizing approach-related morbidity in obese patients.

Accurate clinical staging of CES is paramount for determining prognosis. According to the classification proposed by Gleave and Macfarlane (4), CES is stratified into incomplete CES and complete CES stages based on urinary function. incomplete CES is characterized by sensory deficits or subjective urinary changes but with preserved executive bladder control. In contrast, complete CES presents with painless urinary retention and overflow incontinence. Our patient presented with saddle anesthesia and perineal pain but retained the ability to void voluntarily. This strictly fulfilled the criteria for incomplete CES. Distinguishing incomplete CES from the retention stage is critical. Early decompression in this “incomplete” window leads to significantly better neurological recovery than delayed intervention in the complete stage.

Differentiating the primary pathology in the presence of concurrent spinal degeneration remains a diagnostic challenge. Although our patient presented with lumbar spondylolisthesis and multi-level disc herniation (L1–S1). However, the MRI revealed circumferential compression characteristic of SEL (the “Y-sign”). This is distinct from the ventral impingement typical of disc herniation. The severity of thecal sac constriction at L5–S1 strictly correlated with the patient's perineal symptoms. This clinico-radiological concordance justified our conclusion. We concluded that SEL, rather than the degenerative changes, was the precipitating factor for the acute crisis.

A critical decision in this case was limiting decompression to the L5–S1 level despite the cephalad extension of adipose tissue to L3. This targeted decompression strategy was based on symptomatology. The patient's primary complaints were saddle anesthesia and perineal pain, localizing to the sacral nerve roots. We intentionally avoided prophylactic decompression of asymptomatic proximal levels (L3–L4). This minimized surgical time and preserve spinal stability, particularly given the patient's pre-existing spondylolisthesis.

Technically, the rationale for choosing UBE lies in its distinct advantages over established methods. Traditional open laminectomy often requires extensive paraspinal muscle stripping. It also disrupts the posterior tension band, which risks postoperative instability. This is a critical concern in obese patients biomechanically predisposed to spinal stress (12). Microscopic decompressive laminotomy is minimally invasive but limited by rigid tubular retractors and a restricted visual field. These limitations hinder the thorough removal of adipose tissue. This is especially true for fat extending into the lateral recesses (13). Furthermore, percutaneous endoscopic spine surgery (PESS) is constrained by a coaxial single-port design. In contrast, UBE utilizes independent viewing and working portals (14). This separation allows for the use of larger instruments and higher efficiency in resecting the extensive volume of pathological fat. Crucially, the continuous saline irrigation in UBE acts as a “water-dissection” tool. It gently separates adherent fat from the dura (15, 16). This safety feature is unavailable in “dry” open or microscopic surgeries. Recent systematic reviews have also indicated that while UBE has a learning curve, its overall complication profile is comparable to or superior to microscopic surgery once proficiency is attained (17).

Despite these technical advantages, clinical documentation of UBE for SEL remains scarce. Kang et al. (18) first described this technique. They reported successful outcomes in three patients with claudication or radiculopathy. They used percutaneous biportal endoscopic surgery (PBES). Subsequently, Liu et al. (19) and Li et al. (20) contributed additional case reports confirming the feasibility of UBE for symptomatic SEL, noting significant pain relief and effective fat removal. More recently, comparative and observational studies have provided stronger evidence regarding its safety and efficacy. Tan et al. (21) demonstrated in a cohort of 15 patients that UBE resulted in significantly less blood loss and shorter hospital stays compared to open decompression. Similarly, Gao et al. (22) analyzed 15 patients undergoing UBE-ULBD, confirming significant expansion of the dural sac cross-sectional area with a facet joint preservation rate of 77.8%. However, the existing literature predominantly addresses chronic neurogenic claudication. Reports of UBE specifically for incomplete CES are exceptionally rare. Our case uniquely bridges this gap. It highlights that UBE is not only feasible for elective cases but also safe and effective for urgent neural decompression in high-risk obese patients.

However, this study has limitations, primarily its nature as a single case report, which necessitates further validation through larger cohort studies. In this case, formal follow-ups were conducted at 6 and 12 months postoperatively. At the 12-month mark, the patient remained entirely asymptomatic, maintaining a stable and pain-free gait. This sustained clinical stability, even in the presence of L4 spondylolisthesis, suggests that the targeted UBE decompression successfully maintained spinal integrity. Nevertheless, lifelong monitoring remains essential to definitively assess the risk of late lipomatosis recurrence or degenerative progression at unoperated segments.

Ultimately, early identification and holistic management are critical. We emphasize that the “Y-sign” and high-grade compression on MRI should be interpreted as warning signs. They indicate an impending neurological crisis in high-risk patients (BMI = 29.4 kg/m^2^) (8). Furthermore, long-term success requires addressing the underlying metabolic syndrome (23, 24). Surgical decompression must be paired with metabolic optimization, such as weight reduction and emerging therapies like GLP-1 receptor agonists, to prevent recurrence (25). A multidisciplinary approach is therefore essential. It should combine minimally invasive surgery with metabolic intervention.

## 4 Patient perspective

The patient reported immediate relief from perineal pain and numbness following the surgery. He expressed high satisfaction with the minimally invasive procedure, which allowed for early mobilization and a quick return to daily activities. At the 6-month follow-up, he was grateful for the significant improvement in his quality of life.

## 5 Conclusion

Spinal epidural lipomatosis, though rare, should be considered in the differential diagnosis of incomplete CES, particularly in patients with risk factors such as obesity or chronic steroid use. As illustrated in this case, prompt recognition through lumbar MRI is crucial for timely intervention. The successful application of UBE decompression in this patient highlights its efficacy as a minimally invasive surgical option for SEL-induced incomplete CES, offering significant neurological recovery and functional improvement. The successful outcome in this case aligns with the evolving trend toward endoscopic spine surgery and underscores the importance of tailoring treatment to both the pathology and the patient's clinical profile.

## Data Availability

The datasets presented in this article are not readily available because of ethical and privacy restrictions. Requests to access the datasets should be directed to the corresponding authors.
